# Optimisation of Mesh Enclosures for Nursery Rearing of Juvenile Sea Cucumbers

**DOI:** 10.1371/journal.pone.0064103

**Published:** 2013-05-23

**Authors:** Steven W. Purcell, Natacha S. Agudo

**Affiliations:** 1 National Marine Science Centre, Southern Cross University, Coffs Harbour, New South Wales, Australia; 2 Natural Resources Management, The WorldFish Center, Penang, Malaysia; University of Connecticut, United States of America

## Abstract

Mariculture of tropical sea cucumbers is promising, but the nursery rearing of juveniles is a bottleneck for farming and sea ranching. We conducted four medium-scale experiments lasting 3–6 weeks, using thousands of cultured juvenile sandfish *Holothuria scabra,* to optimise nursery rearing in mesh enclosures in earthen seawater ponds and to test rearing in enclosures in the sea. In one experiment, survival in fine-mesh enclosures (1 m^3^; 660-µm mesh) related nonlinearly to juvenile size, revealing a threshold body length of 5–8 mm for initial transfer from hatchery tanks. Survival in enclosures within ponds in the other experiments ranged from 78–97%, and differences in growth rates among experiments were explained largely by seasonal differences in seawater temperatures in ponds. Stripped shadecloth units within fine-mesh enclosures increased feeding surfaces and improved growth rates by >15%. On the other hand, shading over the enclosures may lower growth rates. Following the rearing in fine-mesh enclosures, small juveniles (0.5 to 1 g) were grown to stocking size (3–10 g) in coarse-mesh enclosures of 1-mm mesh. Sand or mud added to coarse-mesh enclosures did not significantly improve growth compared to controls without sediment. Survival of sandfish juveniles in coarse-mesh enclosures set on the benthos within seagrass beds differed between two sheltered bays and growth was slow compared to groups within the same type of enclosures in an earthen pond. Our findings should lead to significant improvement in the cost-effectiveness of rearing sandfish juveniles to a stocking size compared to established methods and highlight the need for further research into nursery systems in the sea.

## Introduction

Sea cucumbers (Echinodermata, Holothuroidea) have attracted global interest for mariculture and multi-trophic culture systems because of their high market value and ability to thrive on the waste products of fish and shellfish [1]–[3]. They are now cultured at commercial scales in China, Japan, Maldives, Madagascar, Australia, New Caledonia, Palau, Mexico and Vietnam [4]. One temperate species, *Apostichopus japonicus*, is cultured in billions while four tropical species have been produced in hundreds of thousands [4]. Sea cucumbers are firstly cultured in tanks and the newly settled juveniles grow on diatom-covered plates [5]–[7]. They must then be grown in nursery systems to a larger (i.e. fingerling or ‘sluglet’) size for stocking into earthen seawater ponds or into the sea for stock restoration, sea ranching, or farming in sea pens or cages. For tropical species like the sandfish (*Holothuria scabra*), this means to a body weight of 1–20 g for stocking into ponds [8]–[10], or to 3–15 g for culture at sea in pens or for sea ranching [11], [12]. However, a lack of published technology on nursery systems has hindered cost-effective commercial production of juveniles [4].

Sandfish can attract up to US$6 per kg fresh weight (gutted) but their growth to a large commercial size, e.g. 0.7–1 kg, would take at least 2 years in earthen ponds [4], [8], [13] or 3+ years in coastal seagrass beds [11], [12]. Survival of sandfish from released juveniles (3–15 g) to minimum market size (300–500 g) in sea pens or in a sea ranch appears to be generally in the range of 10–30% [4], [11], [12]. To compensate for long grow-out cycles and potentially low survival, sea cucumber juveniles must be produced cheaply to a competent size for stocking [4], [14]. Culture systems for sea cucumbers are relatively labour intensive, especially in the hatchery [7], [15]. Mariculture profitability is therefore predicated on systems that allow juvenile sandfish to be transferred, at an early stage, out of hatchery tanks and into nursery units where they survive well and grow to a size for stocking in a short timeframe [4].

Battaglene [6] and Pitt [16] described the early technology of culturing sandfish larvae and rearing the newly-settled juveniles on stacked plates, or on sand, within tanks. However, competition for space becomes the most significant constraint for grow-out of juveniles to a stocking size, especially where surface area of hatchery tanks is limited. Battaglene et al. [17] found in Solomon Islands that growth of sandfish juveniles is impeded once densities reach 225 g m^−2^ of tank floor space; a threshold corroborated in Vietnam (R. Pitt, pers. comm.) and New Caledonia [7]. Earthen ponds offer expansive nursery area and a natural source of detritus as food but juvenile sandfish survive poorly when placed directly on pond substrates at a small size [18].

Mesh enclosures in the sea or in earthen ponds are one solution for scaling up production of juvenile sandfish because the costs of mesh and water exchange are relatively low [4], [19]. Pitt and Duy [18] conducted the first pioneering work of growing sandfish juveniles in mesh enclosures within earthen ponds. This nursery system involved two steps: small juveniles of a few mm body length from hatchery tanks are grown in fine-mesh enclosures (mesh size ∼450 µm) to about 20 mm body length (almost 1 g body weight), then transferred to coarse-mesh enclosures of 1-mm mesh and grown further to a competent size for stocking into ponds or into the sea.

In the present study, we sought to develop optimal methods for scaling up the production of sandfish juveniles in mesh enclosures within earthen ponds. We used replicated experiments with cultured sandfish juveniles to (1) determine the effects of shading and increased surface area of mesh in fine-mesh enclosures on growth and survival, (2) identify the minimum size at which small juveniles could be transferred from hatchery tanks into fine-mesh enclosures in ponds, (3) compare growth among substrate types in coarse-mesh enclosures, and (4) assess the viability of sea-based mesh enclosures as a nursery system. Our findings provide new technology, which should help to improve cost effectiveness of rearing juvenile sandfish for land-based mariculture, sea farming, sea ranching and restocking. The findings and experimental approach should be valuable for optimising mariculture of other tropical sea cucumbers.

## Materials and Methods

### Production of Juveniles

Sandfish juveniles for all experiments were produced at a hatchery at Saint Vincent (21.926°S, 166.083°E) near Boulouparis, New Caledonia, using established methods [6], [7], [16]. Induced spawnings during summer months comprised >10 male and female broodstock, hence the juveniles were of mixed parentage. Sea cucumber larvae were reared in 1000-l and 2000-l cylindrical conical-bottom tanks where they settled on the tank walls and on coated fibreglass plates after about 10 rearing days. After 4 weeks, newly-settled juveniles were transferred to 7000-l indoor (90% shade greenhouse) raceway tanks where they were fed daily with dried feeds [7]. Different batches of juveniles from 1–20 mm in length were used for experiments in fine-mesh enclosures and coarse-mesh enclosures (also called ‘hapas’ and ‘bag nets’, respectively, *sensu* [18]). We used some newly settled animals directly from the larval tanks for experiments requiring juveniles <10 mm in length.

### General Procedures

We used two types of supple mesh enclosures: 1×1×1 m fine-mesh enclosures made of 660 µm transparent nylon mesh, and 1×1×1 m coarse-mesh enclosures made of 1 mm black Tentex™ mesh (a heavy gauge, plastic-coated mosquito netting). Each enclosure had a mesh floor but no cover (i.e. no top mesh), and presented a sleeve on the upper edge of the side walls with tie-off cords at 1-m intervals. These were set up in earthen seawater ponds of 0.13–1.14 ha next to the hatchery, and held upright by tying to metal posts externally. The ponds we used were owned by the site owner, IFREMER, who gave permission for the facilities. Seawater in ponds was 0.7 to 1.0 m deep and renewed by constant input from a reservoir. Shrimp (*Litopenaeus stylirostris*) were regularly cultured in these ponds for separate studies and some of the present experiments were conducted in a pond with shrimp at low stocking densities of ∼5 ind m^−2^. The ponds therefore had background levels of organic waste from previous shrimp culture.

All mesh enclosures were held approximately 10 cm above the sandy-mud substratum in ponds to mitigate low-oxygen and high-temperature conditions at the sediment interface, which could occur after water stratification from heavy rains [7]. Mesh enclosures in all experiments were placed in haphazard orientation, approximated 1–2 m apart. The upper rim of mesh enclosures was ∼30 cm above the water surface, excluding shrimp from interacting directly with sea cucumbers.

Prior to each experiment, the mesh enclosures were conditioned in the pond for 3–7 days to acquire a natural biofilm and detritus. Although the sea cucumbers fed on the detritus, excessive fouling was routinely brushed off the upper 50 cm of the inside and outside mesh of the enclosures to allow water exchange.

Sea cucumber juveniles from hatchery tanks and raceways were distributed evenly, according to body sizes, into replicate groups held temporarily in buckets unless stated otherwise. We believed that the smaller juveniles (i.e. <10 cm long) for experiments using fine-mesh enclosures would be injured by blotting and weighing out of seawater because these were known to be less robust than larger juveniles [18]. Therefore, we used body length as a measure of initial animal size for the first two experiments with fine-mesh enclosures. Body length was a satisfactory measure of body size for juvenile sandfish since these measurements explained 91% of the variation in weights among individuals (see *Results*). For the juveniles in the fine-mesh enclosures, average initial body lengths of a random sample (*n* = 20) of individuals were measured to ±1 mm using a ruler set under a Petri dish with seawater in which the animals were held. As the animals were disturbed, the measurements are contracted body length. For small juveniles (<1 g in weight), weights of individuals for examining the relationship between body weight and length were measured on a high-precision electronic balance to 0.001 g (Mettler Toledo, Model AG204).

The larger juveniles used for the last two experiments (*Effects of sediment type and sediment depth*; *Enclosures at sea vs pond*) were firstly held together in a bare tank overnight to defecate sediments and detritus. Those groups were then drained and blotted dry on a damp cloth for 30 sec, weighed as a group to ±0.1 g with an electronic balance (Masscal Model NJW-3000), and assigned randomly to coarse-mesh enclosures. For all experiments, we acclimatised juveniles to pond conditions by gradually adding pond water to the buckets over 10 min before placing them into the enclosures. We randomised replicates among experimental units using random number tables or pulling numbered pieces of paper from a hat.

Water parameters within mesh enclosures were measured every 2–7 days at 5 cm above the bottom mesh surface so that the measurements were close to the enclosure floor where sea cucumbers resided most but not corrupted by precipitated detritus or faeces. We measured dissolved oxygen (to ±0.1 mg L^−1^) and temperature (to ±0.1°C) with a YSI™ handheld D.O. meter and salinity (to ±1 ppt) with an Eclipse refractometer.

At the end of experiments, mesh enclosures were removed from the ponds and the contents transferred through a sieve in seawater to collect the remaining juvenile sea cucumbers. The sea cucumbers were counted twice and weighed, as described, as groups to ±0.1 g and/or individual body lengths measured with a ruler to 1 mm; lengths in all cases are contracted body length. The number of juveniles counted in each group was used to calculate proportionate survival and final average body weight. Additional measurements are described later. In the last experiment, where the juveniles had been used in a previous experiment, they were combined into a bare tank, left overnight, then re-randomised into new groups, as described.

### Effects of Increasing Mesh Surfaces and Shading

**Figure 1:**
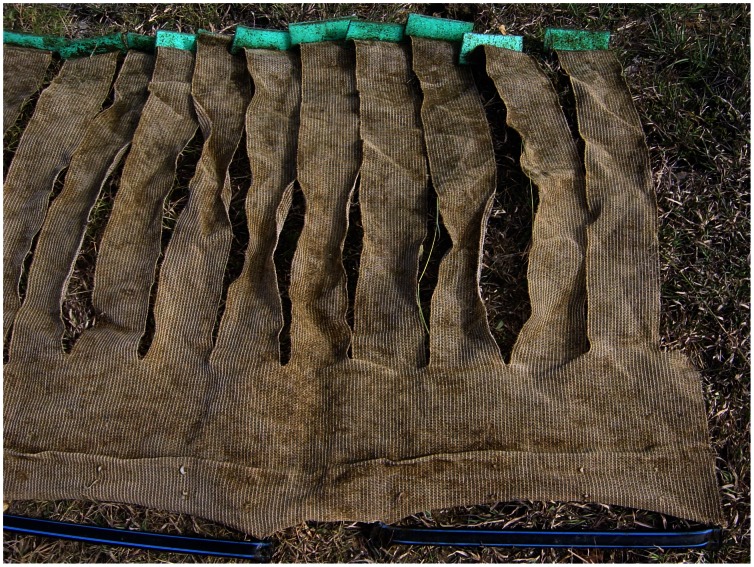
Stripped shadecloth unit. Photograph of the end of a stripped shadecloth unit used to increase the surface area of mesh inside fine-mesh enclosures. The sand-filled polypropylene tubes at the bottom of the photograph weigh down the woven polyethylene fabric while the foam pieces at the top kept the strips upright in the water.

We examined the effects of shade and extra surface area of mesh within fine-mesh enclosures on growth and survival of small juveniles. Sandfish larvae apparently settle onto seagrass blades in the wild and the small juveniles feed on epiphytic matter on the surfaces of the blades until about 6–9 mm in length [20], [21]. In addition, lower light levels have been shown to extend foraging periods of juveniles [22], [23]. We constructed ‘stripped shadecloth’ units using a 3.5 m×0.7 m band of woven coated polyethylene fabric (‘shadecloth’), weighted to the floor of enclosures with sand-filled polypropylene tubes (Fig. 1). Strips of ∼10 cm were cut across ∼80% of the width of the shadecloth and made buoyant at the upper edge with foam, such that the strips mimicked the erect structure of seagrass when set in the enclosures. The units were laid in a zig-zag fashion within the enclosures and acted to increase the surface area that the small juveniles could feed upon. Within fine-mesh enclosures, the units provided 60% more surface area on which detritus could accumulate and sea cucumber juveniles could climb.

**Figure 2:**
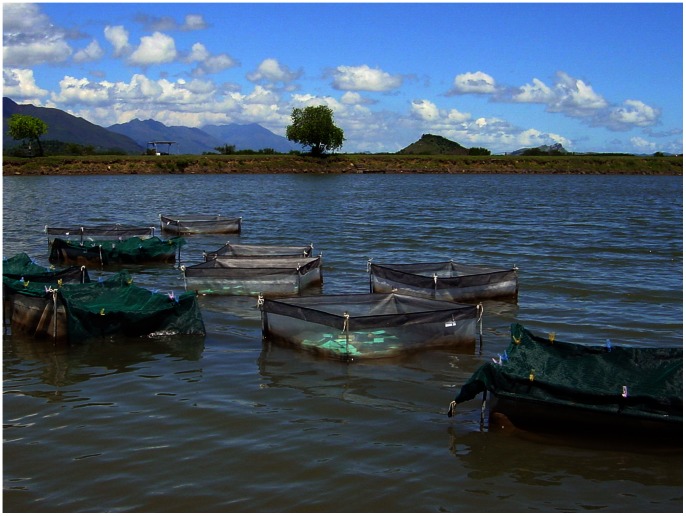
Fine-mesh enclosures. Photograph of 1-m^3^ fine-mesh enclosures (660 µm mesh) in the earthen pond during the first experiment (Effects of increasing mesh surfaces and shading), showing some replicates shaded with dark shadecloth and some with stripped shadecloth inside.

During summer, 24 fine-mesh enclosures were set in one pond (‘pond H’) then assigned randomly to combinations of two orthogonal factors: shade (a single layer of 70% shadecloth on top of fine-mesh enclosures; two levels: with and without) and stripped shadecloth (two levels: with and without), giving six replicates per combination (Fig. 2). We conditioned one stripped shadecloth unit in each of 12 fine-mesh enclosures for 3 days prior to the experiment. Recently-settled juvenile sea cucumbers for this experiment had not been used in previous experiments. Initial body lengths of the juveniles ranged from 4 to 13 mm, and the average was 6 mm (±2 mm s.d.) based on measurement of a single random sample (*n* = 20) of individuals, as described. This average body length converts (see *Results*) to approximately 0.05 g in weight. We then prepared 24 groups of 200 juveniles, i.e., 4,800 juveniles total. We distributed the larger and smaller individuals as evenly as possible among the 24 groups before randomly allocating and transferring the groups to the replicate fine-mesh enclosures. We did not weigh each group at the start of this experiment, for reasons explained earlier. During this 3-week experiment, juveniles fed only on the natural detritus and fouling in the fine-mesh enclosures. Daily measurements of daytime water temperature averaged 31.6°C, range: 30–33°C, and dissolved oxygen averaged 5.2 mg l^−1^. At the end of the 3 weeks, the number of juveniles surviving in each group was counted and each group was weighed, as described.

### Juvenile Size for Transfer to Fine-mesh Enclosures

We aimed to determine the minimum size at which small sea cucumber juveniles could be transferred from hatchery tanks to fine-mesh enclosures in ponds, based on their survival across groups of varying starting size classes. During summer, 20 fine-mesh enclosures were set up in one pond (‘pond H’). Based on the first experiment (*Effects of increasing mesh surfaces and shading*), each fine-mesh enclosure contained a 3.5 m×0.7 m stripped-shadecloth unit but no cover (top mesh) for shade.

These were different juveniles from the experiment on *Effects of increasing mesh surfaces and shading* and had not been used in other experiments. Recently settled and small juvenile sea cucumbers averaging 1.3–18.2 mm in length were placed into 20 groups of 200 individuals of similar length; i.e. each group represented a different length class. Random samples of 15 individuals from each replicate group were transferred to Petri dishes with seawater temporarily for measuring body lengths, as described. The average precision (SE/mean) of initial body lengths within groups was 6%. We did not weigh each group at the start of the experiment, as explained earlier.

The 20 groups of juveniles were allocated randomly to the 20 fine-mesh enclosures and transferred into them. Again, feed was not added to the fine-mesh enclosures and the juveniles fed solely on natural detritus fouling. After 3 weeks, juveniles were collected, taking care to check the stripped shadecloth units. Juveniles surviving in each group were counted and the groups were weighed, as described. We also measured the lengths of 15 randomly chosen sea cucumbers from each group. Daytime water temperatures averaged 29.6°C and dissolved oxygen in the fine-mesh enclosures was 6 mg l^−1^ throughout the experiment.

### Effects of Sediment Type and Sediment Depth

**Figure 3:**
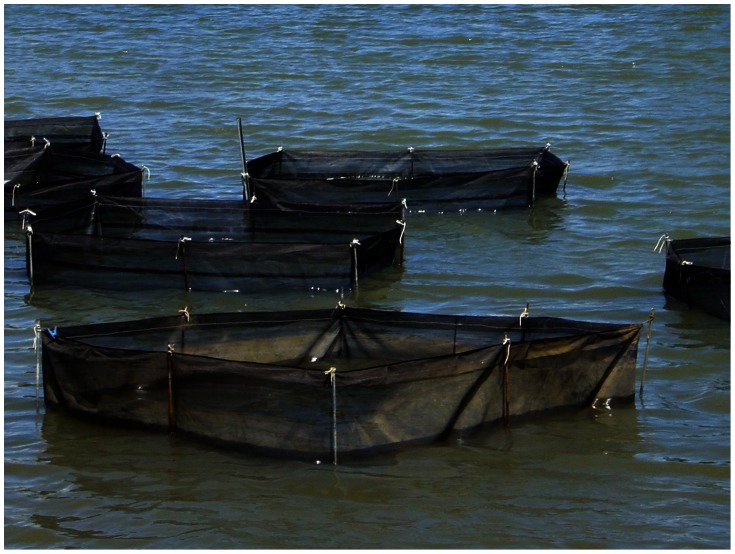
Coarse-mesh enclosures. Photograph of some 2 m×2 m×1-m-high coarse-mesh enclosures (1-mm mesh) in an earthen pond.

This two-factor experiment during winter examined differences in growth and survival of larger juveniles in coarse-mesh enclosures (Fig. 3) among treatments with different quantities of added sediment, either sand or mud. Into one pond (‘pond G1’), we set 24 coarse-mesh enclosures of 1 m^2^ (floor area) directly onto the pond floor in haphazard orientations. Treatment combinations of sediment type (two levels: river *sand*, averaging 325 µm grain size and conditioned with Algamac 2000™; and *mud*, collected from the pond floor) and sediment depth (three levels: 1, 3, and 5 mm, distributed by volume) were then allocated randomly to coarse-mesh enclosures (*n* = 3), leaving six coarse-mesh enclosures without sediment as controls.

We used juveniles from the hatchery that had not been used in other experiments. One week after the coarse-mesh enclosures were set up with their respective treatments, juveniles averaging 0.56 g in weight (range: 0.3–1.1 g) were allocated into 24 groups of 50 individuals. We took care to homogenise size distributions among groups before the random allocation to experimental units and the range in group weights was 23–32 g. We weighed each group (of 50 animals) separately to ±0.1 g, as described earlier, and used initial group weight as a covariate to test potential bias of differences in initial groups’ weights on growth performance. The groups were weighed, allocated randomly to the coarse-mesh enclosures and juveniles transferred into each, as described. No food was added to the coarse-mesh enclosures. We allowed the experiment to run for 6 weeks before collecting, counting and weighing the juveniles in groups, as described.

### Enclosures at Sea vs. Pond

In autumn, we aimed to compare growth and survival of juvenile sea cucumbers over 5 weeks in coarse-mesh enclosures in an earthen pond with those placed in the sea within sheltered bays. We firstly conditioned 4 replicate coarse-mesh enclosures of 1 m^2^ (floor area) for two days in an earthen pond (‘pond G2’) and in each of two shallow subtidal (∼0.3 m depth at zero tidal datum) seagrass beds at Ouano (21.845°S, 165.812°E) and at Baie de Saint-Vincent (21.925°S, 166.080°E), New Caledonia. The corners of the coarse-mesh enclosures were staked to the natural substratum. Due to a 1–1.5 m tidal flux, we gathered and bound the upper part of coarse-mesh enclosures in bays to exclude entry of predators and suspended that part in the water by an attached float.

Small individuals from the *Juvenile size for transfer to fine-mesh enclosures* experiment were used for this experiment after 8 days of being mixed together and fed in a raceway tank to recover and one night together in a bare tank to expel sediments. A total of 1,200 juveniles averaging 0.38 g body weight, ranging from 0.15 to 1 g (equivalent to 10 to 26 mm in length), were distributed evenly by body weight into groups of 100 individuals. We individually weighed 10 individuals sampled randomly from each replicate group at the start of the experiment. Each replicate group of 100 juveniles was weighed separately, prior to randomisation among replicates and treatments, as described, showing a range in group weights from 36–39 g. We then randomly assigned groups to the replicates and placed them into the coarse-mesh enclosures on the same day. No sediment or food was added to the coarse-mesh enclosures, so juveniles only had the naturally accumulated detritus upon which to feed. The upper 50 cm of mesh on all coarse-mesh enclosures were brushed cleaned once, half-way through the experiment to remove excess detritus fouling on the mesh walls to promote water exchange in the enclosures. At the completion, the coarse-mesh enclosures were retrieved and the juveniles surviving in each group were counted and groups were weighed, as described.

### Statistical Analyses

We used Levene’s test to examine homogeneity of variances of data from the experiments, and transformed data as appropriate (specified below). For the experiment on *Effects of increasing mesh surfaces and shading*, we compared proportionate survival between levels in treatments (with or without shade; with or without stripped shadecloth; fixed factors) using a two-way ANOVA on arcsine √y transformed data. Differences in final mean individual weight, water temperature and oxygen concentration between treatment levels were tested using two-way ANOVA tests. Mean final individual weight per group (i.e. total final group weight divided by total number surviving, for each group) and survival per group were used as the data for analyses. Heteroscedastic data (*P* = 0.012) of final mean individual weight should not be problematic since the experiment was balanced with sufficient replication [24].

For the experiment on *Juvenile size for transfer to fine mesh enclosures*, we examined the relationship between survival and the juvenile size (body length) for transfer of sea cucumbers to fine-mesh enclosures using nonlinear regression modelling. Mean initial body length of individuals per group (i.e. averages of the measurements of 15 sampled animals from each of the 20 groups) and the survival per group were used as the data for analyses (*n* = 20). We fitted the data to 298 standard functions using DataFit 8.0 software (Oakdale Engineering) and used Akaike’s Information Criterion (AIC) to determine the most appropriate function [25].

For the experiment on *Effects of sediment type and sediment depth*, two-way orthogonal analysis of covariance (ANCOVA) tests were used to compare growth (mean individual weight gain) and survival among substrate type and substrate depths using initial mean body weight within each group as the covariate. Mean weight gain of animals (i.e. total group weight divided by total number surviving, minus the initial mean individual starting weight for each group) and survival per experimental unit (enclosure) were used as the data for analyses (*n* = 3; *N* = 18). Based on non-significant difference in mean growth of juveniles among substrate depths, we then pooled data among substrate depths within both substrate types (*n* = 9) in order to compare mean individual weight gain of juveniles with that of control groups (*n* = 6) using a one-way ANCOVA, with the same covariate. ANCOVA is essentially an ANOVA on data adjusted by the regression slope of the response variable on the covariate (e.g., measurements of an initial condition of replicates) and provides a test of the covariate’s effect on the response [26]. In this study, the ANCOVA tests accounted for the potential effect of variations in initial mean animal weight among groups on their subsequent survival and average growth.

For the last experiment on *Enclosures at sea vs pond*, we used one-way ANCOVA tests to compare growth and survival of juvenile sea cucumbers between locations at sea and in a pond, using initial mean body weight as a covariate. Mean weight gain of animals (i.e. total final group weight divided by total numbers surviving, minus the initial mean individual starting weight for each group) and survival per experimental unit (enclosure) were used as the data for analyses (*n* = 4; *N* = 12). For those tests, we used untransformed data since errors were unconstrained [24] and data were homoscedastic.

## Results

### Length and Weight Measurements

Precise measurements (lengths ±1 mm and weights ±0.01 g) of 303 juveniles, ranging from 6 to 70 mm in body length, from various experiments provided growth functions for converting measurements of length to weight and vice versa:
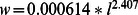


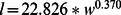
where: *l* is length in mm and *w* is weight in g.

**Figure 4:**
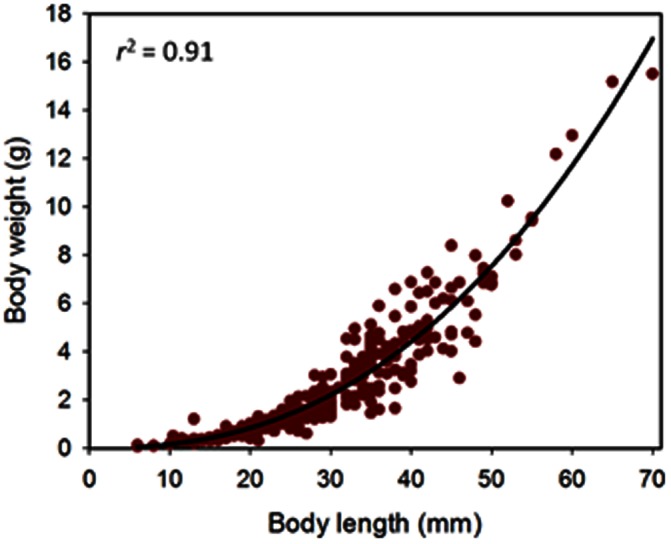
Body length vs body weight of juveniles sandfish. Scatterplot shows relationship (growth curve) of body length with body weight for sandfish juveniles of 6–70 mm in length. Juveniles were drained and blotted dry on a damp cloth for 30 sec before being measured and weighed.

Body length provided a reasonable prediction (*F*_1, 301_ = 2877, *P*<0.001, *r* ^2^ = 0.91) of the drained body weight of these hatchery-produced juveniles (Fig. 4). The scaling coefficient of 2.407 shows negative allometric growth in sandfish; i.e. the juveniles become more slender (weigh relatively less for their length) as they grow and increase in body length.

### Effects of Increasing Mesh Surfaces and Shading

**Figure 5:**
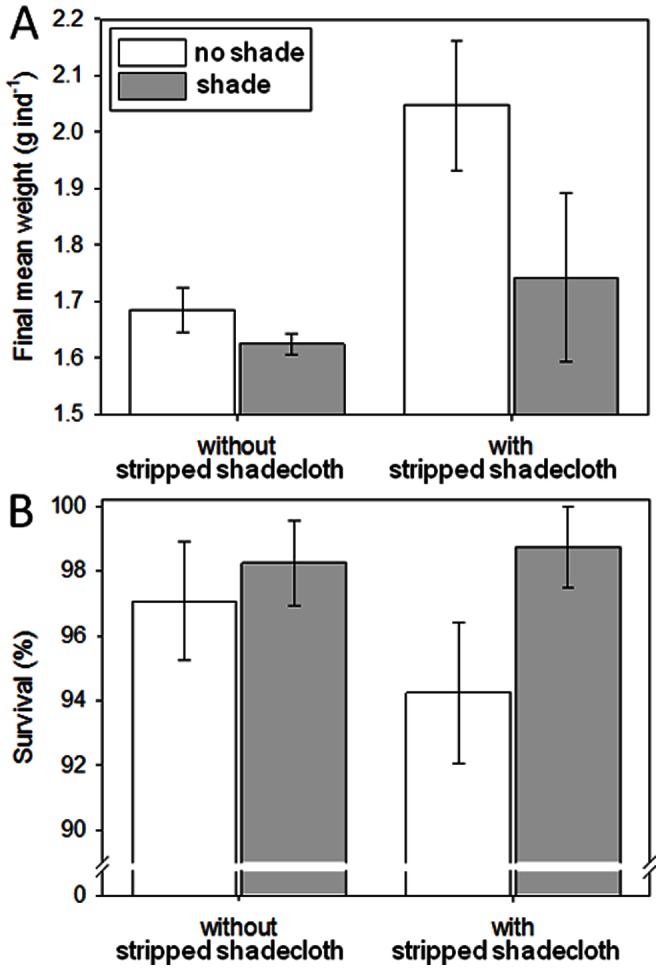
Effects of increasing mesh surfaces and shading. Bar graphs of (A) average individual body weights and (B) average survival of sandfish in fine-mesh enclosures with and without stripped shadecloth, and with (shaded) and without (open) shade; *n* = 6. Error bars are 1 standard error of the mean.

Survival averaged 97% and was similar between levels of both main effects and the interaction (*F*-values = 0.51–2.89, *P*-values = 0.11–0.48). Since this experiment was in mid-summer, growth of the juveniles was rapid for their body size. Final mean individual weights among treatments averaged 1.6 to 2.0 g, which are greater than needed for transfer to coarse-mesh enclosures. Considering the converted initial individual average body weight of 0.05 g, average growth rates equate to 0.07–0.10 g day^−1^. Final body weights of sandfish juveniles were more variable in fine-mesh enclosures with stripped shadecloth (Fig. 5). Mean final body weight was significantly higher in the fine-mesh enclosures with stripped shadecloth than those without (*F*_1, 20_ = 6.22, *P* = 0.02). Juveniles in unshaded fine-mesh enclosures attained marginally non-significantly greater weights than those in shaded fine-mesh enclosures (*F*_1, 20_ = 3.60, *P* = 0.07). The stripped shadecloth improved growth of sandfish juveniles by more than 15% compared to fine-mesh enclosures without them.

Seawater temperatures in the pond were high (30–33°C) and water parameters were consistent within treatments but different between them (Table 1). Shade lowered the average water temperatures by 0.14–0.18°C in fine-mesh enclosures and this effect was statistically significant (*F*_1, 20_ = 96.6, *P<*0.001) and consistent between fine-mesh enclosures with and without stripped shadecloth (interaction: *F*_1, 20_ = 1.79, *P* = 0.20). Dissolved oxygen in fine-mesh enclosures averaged 0.24–0.45 mg l^−1^ higher in unshaded fine-mesh enclosures than shaded fine-mesh enclosures (*F*_1, 20_ = 28.0, *P<*0.001). The average dissolved oxygen concentrations were 0.84–1.06 mg l^−1^ higher without stripped shadecloth than with (*F*_1, 20_ = 208.8, *P<*0.001), and the interaction of main effects was non-significant (*F*_1, 20_ = 2.58, *P* = 0.12).

**Table 1:** Average water parameters in fine-mesh enclosures within the pond in the first experiment (*Effects of increasing mesh surfaces and shading*).

	Without stripped shadecloth		With stripped shadecloth	
	Without shade	With shade	Without shade	With shade
Temperature (°C)	31.8±0.1	31.6±0.1	31.7±0.1	31.6±0.1
Dissolved oxygen (mg l^−1^)	5.9±0.1	5.4±0.1	4.8±0.1	4.6±0.1

Error estimates are standard errors of averages for the four treatment combinations.

### Juvenile Size for Transfer to Fine-mesh Enclosures

**Figure 6:**
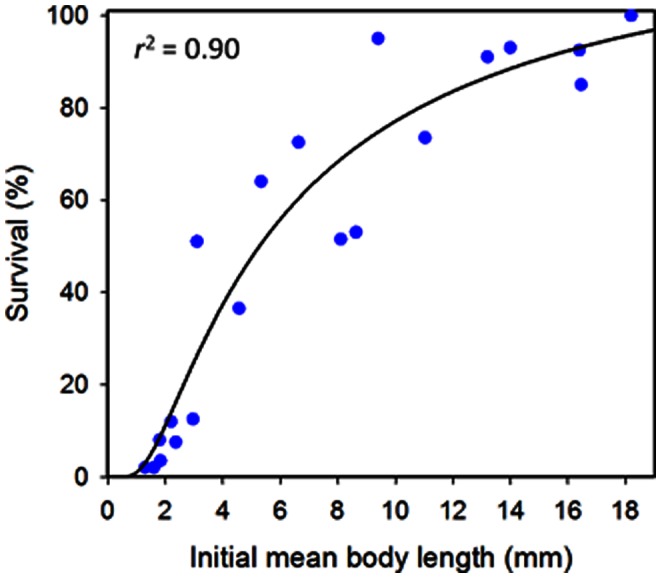
Effects of sediment type and sediment depth. Scatterplot of survival of juvenile sandfish in fine-mesh enclosures across various initial mean group body lengths, three weeks after transfer from hatchery tanks; *n* = 20 fine-mesh enclosures. The fitted curve is the most appropriate nonlinear function by AIC selection.

The recovery rate (survival) of juvenile sea cucumbers from the fine-mesh enclosures was highly variable among groups and related significantly to initial mean body lengths of the animal groups (*F*_1, 18_ = 168.3, *P*<0.001) (Fig. 6). The modelled relationship (*r* ^2^ = 0.90) suggests that more than 50% of juveniles longer than 5 mm at initial transfer could be expected to be recovered from similar fine-mesh enclosures after 3 weeks.

Growth rates, using initial lengths converted to weights, were highly variable among groups, due to the wide range in initial sizes. Growth averaged 0.022 g ind^−1^ day^−1^ for groups of juveniles with mean starting lengths >5 mm. The sandfish juveniles increased their body weights by 2 to 35 times their initial body weights (converted from body length) over the 21 days.

### Effects of Sediment Type and Sediment Depth

Seawater temperatures were low in the pond (mean: 21.3°C) because this experiment was conducted in winter and, consequently, growth of the sandfish was slow relative to previous experiments. From a starting individual average weight of 0.56 g, juveniles averaged only 1.13 g after six weeks. Across all treatments, the average growth rate was 0.013 g ind^−1^ day^−1^. Survival averaged 78%. There were no significant differences in growth of juveniles between substrate types (*F*_1, 11_ = 0.26, *P* = 0.62), nor among substrate depths (*F*_2, 11_ = 1.78, *P* = 0.21), nor the interaction between the two factors (*F*_2, 11_ = 0.78, *P* = 0.48). The initial group weights (the covariate) did not significantly affect the differences in growth among replicate groups (*F*_1, 11_ = 0.04, *P* = 0.85). Likewise, neither substrate type nor depth affected survival of juveniles (*F*-values = 0.13–0.96, *P*-values = 0.41–0.73) and initial group weights had no significant effect on survival (*F*_1, 11_ = 0.19, *P* = 0.68). After pooling the non-significant term of substrate depths, to enable a one-way ANCOVA test to include control groups (no sediment), we found no significant differences in growth rates of juveniles with sand or mud compared to juveniles with no sediment (*F*_2, 20_ = 1.88, *P* = 0.18).

### Enclosures at Sea vs. Pond

**Figure 7:**
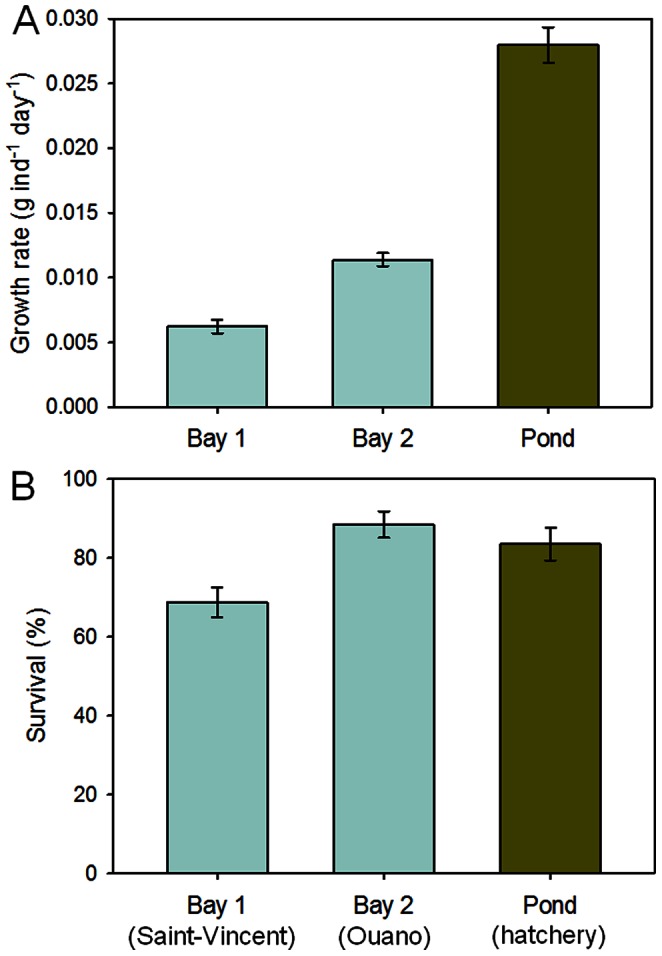
Enclosures at sea vs. pond. Bar graphs comparing (A) average growth rates and (B) average survival of sandfish in coarse-mesh enclosures at two sites at sea and those in an earthen pond; *n* = 4. Error bars are 1 standard error of the mean.

The average individual growth of sandfish juveniles differed significantly among the three locations (*F*_2, 8_ = 176, *P*<0.001) (Fig. 7) and the effect of starting weight on growth was non-significant (covariate: *F*_1, 8_ = 2.9, *P* = 0.13). Growth varied little among the replicate enclosures within locations and differed between the two sites at sea and was markedly low in both compared to the other experiments (average growth rates: 0.006 and 0.011 g ind^−1^ day^−1^, respectively). Growth of juveniles in coarse-mesh enclosures within the pond was significantly higher than those at sea, but still relatively low (average growth rate: or 0.028 g ind^−1^ day^−1^).

Survival differed significantly among locations (*F*_2, 8_ = 5.72, *P* = 0.03) (Fig. 7) and was not affected significantly by starting weight of the juveniles (covariate: *F*_1, 8_ = 0.25, *P* = 0.63). Average survival of juveniles in coarse-mesh enclosures at sea at Ouano (89%) was similar to that of juveniles in coarse-mesh enclosures within the earthen pond at the hatchery (84%), but survival was much lower in coarse-mesh enclosures at sea at Saint-Vincent (69%). Encouragingly, the coarse-mesh enclosures at sea were undamaged and prevented the entry of predators.

## Discussion

Mesh enclosures in ponds are a cost-efficient nursery system for other marine animals [27]–[29]. They are cheaper than tanks, and aquaculture ponds offer natural productivity of organic detritus and cheap supply of water under relatively stable conditions for rearing sandfish juveniles.

Recently, fine-mesh enclosures have been used in Vietnam as the sole unit for growing sandfish juveniles to a competent size for stocking [30]. However, cleaning of fouled fine-mesh enclosures incurs high labour costs and fouling reduces water flow, decreasing the availability of natural food and water quality inside fine-mesh enclosures [27]. Our two-stage nursery system should provide better water exchange by using coarse-mesh enclosures as a second stage, reducing the need for frequent cleaning, but at the expense of additional materials and transfer of juveniles. Regardless of the approach, our experimental findings should be invaluable to increasing the cost-effectiveness of production. Our experiment on *Effects of increasing mesh surfaces and shading* in enclosures furnishes an innovation that should provide 15% faster growth rates of newly-transferred juvenile sandfish, which should reduce the time, and cost, of producing larger juveniles for farming, sea ranching or restocking.

### Length and Weight Measurements

Body length was a relatively good predictor of body weight of hatchery-produced sandfish juveniles in this study. The result may be surprising, considering the previous finding that length explained 61% [31] and 79% [32] of the variation in body weights of adult sandfish and 73% of the variation in weights of sandfish juveniles [17]. We used body length as an initial measure of the size of sandfish in the first two experiments (in fine-mesh enclosures) to avoid disproportionate stress on very small juveniles from the out-of-water weighing procedures.

While adult sandfish have deep transverse wrinkles in their body [33] and can contract like an accordion, juveniles lack such wrinkles. Despite the lack of concurrence with findings of Battaglene et al. [17], we posit that body weight may be predicted from body length more accurately in sandfish juveniles than adults. A close relationship between the two measures (*r* ^2^ = 0.95) was also found for hatchery-produced *Holothuria fuscogilva* juveniles [34]. Indeed, body length is often used as a convenient descriptor of size of small (<1 g) juvenile sea cucumbers in hatcheries [10], [17], [21], [35]–[37].

### Early Nursery of Small Juveniles

We devised the stripped shadecloth units as a means of increasing surface area of mesh in the fine-mesh enclosures on which the juveniles could feed and to increase surfaces on which microalgae and detritus could accumulate. Newly-settled sandfish can be found on seagrass blades in the wild and appear to begin living on the sediment surface at around 6–9 mm in length [20], [21]. Our finding of significantly better growth of sandfish juveniles in fine-mesh enclosures with stripped shadecloth units indicates that the animals either received more detrital food matter with these units and/or foraged better when provided with more surface area of mesh. This innovation should markedly improve the rate of growing small juvenile sandfish of 5–10 mm in length (c.a. 0.03–0.16 g) to sizes of about 1.0 g (22 mm). For early nursery rearing of sandfish juveniles of 0.05–1 g, the growth rates of 0.1 g ind^−1^ day^−1^ that we found for juveniles in unshaded fine-mesh enclosures with stripped shadecloth are comparable to growth rates of similar-sized sandfish in the Philippines within mesh enclosures in a pond ([10]; average: 0.07–0.18 g ind^−1^ day^−1^) and in floating enclosures at sea ([19]; range: 0.004–0.12 g ind^−1^ day^−1^). We did not examine whether the stripped shadecloth could continue to improve growth of sandfish juveniles larger than 1 g because they generally climb less on upright surfaces [17] but that could be a useful subject for further research.

Studies on the diurnal burying cycle and effect of light on the surface foraging behaviour of sandfish juveniles [20], [22], [23] suggested that they may feed over longer periods and, hence, grow faster in shaded tanks. On the other hand, growth rates and survival of sandfish juveniles have been shown to decline in shaded compared to unshaded tanks [17]. We found that shading did not improve the growth of sandfish juveniles in fine-mesh enclosures and slowed growth rates, although the effect was marginally non-significant.

### When to Transfer of Juveniles from Hatchery Tanks to Mesh Enclosures?

Early transfer of small juveniles from hatchery tanks to less resource-intensive nursery systems reduces production costs but sandfish juveniles do not seem to handle being transferred to pond conditions at very small sizes [18]. Our modeling of survival across size classes of sandfish juveniles suggests that the animals should be 5–8 mm body length before being transferred from hatchery tanks to fine-mesh enclosures in ponds. Above this threshold, the survival rate is similar or better than in nursery tanks in the hatchery [7], [15], [18].

A large proportion of the smallest sea cucumbers (<5 mm in length) unrecovered from the fine-mesh enclosures may have conceivably escaped by squeezing through the mesh. Such escapement could have biased our growth rate estimations, since the starting weight of larger remaining individuals would change the calculated weight gain. However, this potential bias would be relatively small and was minimized in our experiment on *Juvenile size for transfer to fine-mesh enclosures* by precise (6% variation) size classes and we do not report growth rates of the smallest size classes. Finer mesh enclosures of 450 µm used in the nursery of sandfish juveniles in Vietnam [18] could reduce or prevent escapement of juveniles of just a few mm in length. However, such mesh was unavailable to us at the time of our study and we considered that water exchange in the fine-mesh enclosures would become impeded with finer mesh. In view that Gamboa et al. [10] found low escapement rates (as low as 4%) of small juvenile sandfish (some just 3 mm in length) from some enclosures of larger (1-mm) mesh, mortality of our small juveniles may be the more likely explanation of low recovery rates from our fine-mesh enclosures than escapement.

### Nursery to Stocking Size

Once cultured sandfish reach about 1 g in weight, a behavioral change finds them preferring, and growing better on, sandy or muddy-sand substrates [17]. Juvenile sandfish in the wild appear to need muddy-sand in which to bury, presumably to avoid diurnal predators [22], [23]. We found no benefit in growth or survival of sandfish juveniles between coarse-mesh enclosures with sand or pond mud and no sediment, nor among different sediment depths. It is possible that sand or pond mud could have improved growth significantly had the experiment been conducted in summer, when growth was faster. Indeed, Juinio-Meñez et al. [19] reported higher weight gain of sandfish juveniles in tanks with sand than in bare tanks, although methods and results were not presented. Our finding suggests that, at least for cultured sandfish in New Caledonia, sand may not be needed for, and does not improve, the assimilation of organic detritus. However, from an ecological perspective, providing juvenile sandfish with sand in the couple weeks before stocking into the sea may still serve as ‘behavioural conditioning’ to encourage diurnal burying, which could conceivably improve predator avoidance and post-release survival rates [19], [23].

### Nursery Systems at Sea?

In cases where suitable earthen ponds are unavailable, the nursery rearing of juvenile sea cucumbers might need to be in the sea. Unfortunately, 1-mm mesh coarse-mesh enclosures gathered at the top and attached to the benthos do not appear to be a viable nursery system (Fig. 7). The slow growth rates of sandfish juveniles that we measured in replicate coarse-mesh enclosures at both protected bays would stifle production of juveniles for stocking programs. Fouling on the coarse-mesh enclosures in seagrass beds at sea may have prevented a sufficient supply of detritus to juveniles or the inside of the coarse-mesh enclosures was too shaded because they were gathered and tied at the top. The mediocre survival of sandfish juveniles in coarse-mesh enclosures at one of the bays further suggests that nursery systems in the sea may be riskier than in ponds. Recently, Juinio-Meñez et al. [19] tested different enclosure systems in the Philippines for rearing sandfish juveniles starting at 4–10 mm over 1–2 months and also found poor survival (18%) of juveniles in enclosures set on the sea bed compared to a floating enclosures (12–44%) or enclosures in an earthen pond (57–73%). They reported modest growth rates in the floating enclosures of up to 0.018 g ind^−1^ day^−1^ in one trial and a range in individual growth of 0.003–0.118 g ind^−1^ day^−1^ in a second experiment. Further research needs to examine the design and maintenance of such floating enclosures to improve growth rates. If mesh enclosures at sea is the only viable nursery strategy, sites should also be evaluated experimentally to find ones that give good growth and survival of the juveniles.
